# Modulation of Mechanosensitive Potassium Channels by a Membrane-targeted Nongenetic Photoswitch

**DOI:** 10.1021/acs.jpcb.3c04551

**Published:** 2023-10-10

**Authors:** Matteo Moschetta, Vito Vurro, Valentina Sesti, Chiara Bertarelli, Giuseppe Maria Paternò, Guglielmo Lanzani

**Affiliations:** † Center for Nano Science and Technology, Istituto Italiano di Tecnologia, Via Rubattino, 81, 20134 Milano, Italy; ‡ Department of Chemistry, Materials and Chemical Engineering “Giulio Natta”, Politecnico di Milano, Piazza Leonardo da Vinci 32, 20133 Milano, Italy; § Department of Physics, Politecnico di Milano, Piazza Leonardo da Vinci 32, 20133 Milano, Italy

## Abstract

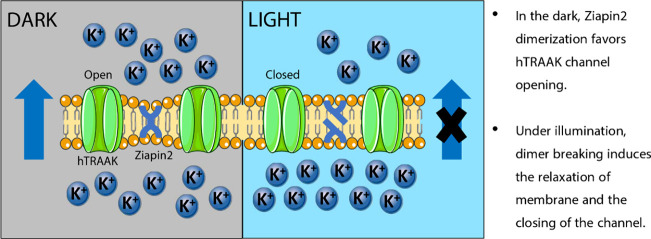

Mechanosensitive ion channels are present in the plasma membranes of all cells. They play a fundamental role in converting mechanical stimuli into biochemical signals and are involved in several physiological processes such as touch sensation, hearing, and blood pressure regulation. This protein family includes TWIK-related arachidonic acid-stimulated K^+^ channel (TRAAK), which is specifically implicated in the maintenance of the resting membrane potential and in the regulation of a variety of important neurobiological functions. Dysregulation of these channels has been linked to various diseases, including blindness, epilepsy, cardiac arrhythmia, and chronic pain. For these reasons, mechanosensitive channels are targets for the treatment of several diseases. Here, we propose a new approach to investigate TRAAK ion channel modulation that is based on nongenetic photostimulation. We employed an amphiphilic azobenzene, named Ziapin2. In the dark, Ziapin2 preferentially dwells in the plasma membrane, causing a thinning of the membrane. Upon light irradiation, an isomerization occurs, breaking the dimers and inducing membrane relaxation. To study the effect of Ziapin2 on the mechanosensitive channels, we expressed human TRAAK (hTRAAK) channels in HEK293T cells. We observed that Ziapin2 insertion in the membrane is able per se to recruit hTRAAK, permitting the exit of K^+^ ions outside the cells with a consequent hyperpolarization of the cell membrane. During light stimulation, membrane relaxation induces hTRAAK closure, generating a consistent and compensatory depolarization. These results add information to the Ziapin2 mechanism and suggest that membrane deformation can be a tool for the nonselective modulation of mechanosensitive channels.

## Introduction

Mechanical stimuli play a pivotal role in maintaining cellular homeostasis and modulating cell signaling in both pathology and physiology.^1−8^ In cells, such stimuli are converted into electrical signals by mechanosensitive ion channels.^9−11^ All types of cells, including both prokaryotes and eukaryotes, express specific mechanosensitive channels, particularly excitable cells like neurons.^12−14^ In the mammalian central nervous system, neurons specifically express TWIK-related arachidonic acid-activated K^+^ channels (TRAAK channels),^13,15^ which are selective for potassium ions and are members of the two-pore domain K^+^ channels (K2P).^16,17^ Under physiological conditions, TRAAK channels are responsible for generating leak currents that regulate and maintain resting membrane potential.^18^ TRAAK channels are also implicated in regulating a variety of important neurobiological functions, such as neurite migration, neurotransmission, signal transduction, and the saltatory conduction of action potentials at the level of the nodes of Ranvier.^16,19,20^ In the absence of mechanical stimuli, TRAAK channels are basically closed, displaying a low open probability with less than 1% of channels opened. TRAAK channel opening is generated by a conformational change in the protein structure which is energetically favored by membrane lateral tension.^21−24^

In the last decades, K2P channels have been shown to be also implicated in several pathological conditions,^3^ including neurodegenerative diseases and retina photoreceptor degenerations like retinitis pigmentosa (RP). With regard to the former, these include a wide variety of devastating conditions characterized by a gradual and irreversible loss of function in either the central or peripheral nervous system with no resolutive cure.^25^ Starting from the 1960s, several drugs have been tested to treat neurodegenerative diseases with small improvements. Transcranial magnetic stimulation and cell-based therapies have been proposed with poor results.^26^ With regard to the latter, retinal ganglion cells (RGCs) of rd1 mice (an animal model that presents an early onset severe retinal degeneration) display a high level of both TREK-1 and TRAAK channels as a protective mechanism to compensate neuronal hyperexcitability and protect the retina from excitotoxicity.^27^ Therefore, mechanosensitive channels have become therapeutic targets for neurodegenerative disease treatment.^28^

Ion channels can be controlled using specific drugs and electrical currents by means of electrodes or light. Light has several advantages such as high spatial resolution, low invasiveness, and remote control. The use of light to trigger and interrogate cellular activities beyond the use of drugs became popular with the introduction of optogenetics.^29^ Optogenetics is based on genes coding for light-activated ion channels or pumps that are introduced into the nervous system using specifically designed viral vectors.^30−33^ However, inserting an exogenous DNA segment via a viral vector has important drawbacks, especially for adoption in human patients, such as immune responses and delivery issues.^34^ A valid and less invasive alternative is represented by nongenetically encoded photoactuators,^35,36^ which reside into or decorate the cell membrane without any genetic manipulation or covalent bonding.^37,38^ Photoactuators are able to modulate the cell membrane potential through electrophysiological parameters, such as membrane resistance, capacitance, or surface charge by converting light into electrical, mechanical, or thermal stimuli.^39−46^

In this study, we use as an intramembrane light actuator the amphiphilic azobenzene molecule Ziapin2. Ziapin2 is an alkyl-substituted 4,4′-diaminoazobenzene terminated with two pyridinium, with a noncovalent affinity to the cell membrane.^47^ It inserts in the cell membrane, persisting in a *trans* configuration. The insertion in the membrane and the consequent formation of Ziapin2 dimers lead to a thinning of the membrane and an increase of the cell capacitance.^48^ Visible light pulses induce *trans*-to-*cis* isomerization that leads to the breaking of Ziapin2 dimers, with a significant perturbation of the cell membrane potentials.^47,49^ Specifically, during illumination, membrane relaxation generates a rapid hyperpolarization followed by slight depolarization after the end of the stimulus.^50−52^ In the present work, we expressed human TRAAK (hTRAAK) channels into HEK293T (Human Embryonic Kidney) cells via transfection, observing that Ziapin2 insertion in the membrane is able *per se* to recruit part of the hTRAAK channels permitting the exit of K^+^ ions outside the cells. K^+^ outward flux generates a sustained increase in the current and consequently a 2-fold hyperpolarization of the cell membrane. During light stimulation at 474 nm, membrane relaxation induces the hTRAAK channel closure, generating a consistent drop in membrane polarization. These results add information to the intramembrane Ziapin2 working mechanism and suggest that the photoinduced membrane deformation can be a tool for the nonselective modulation of mechanosensitive channels.

## Results

### hTRAAK Channels Do Not Affect Ziapin2 Functioning

We preliminarily assessed the functionality of hTRAAK channels expressed in HEK293T cells through a pIRES:hTRAAK vector. We recorded HEK293T cell currents in whole-cell voltage clamp configuration by applying a ramp protocol ranging from −120 to 60 mV as previously reported (Figure S1).^53^ Transfected cells were recognized by checking the reporter protein fluorescence (Figure S1a). After the recordings of basal ramp currents, we acutely added in the extracellular solution 10 μM of ML 67–33, a mechanosensory channel activator (Figure S1b).^53^ As reported in Figure S1c, Ctrl cells showed a slight but not significant increase of current amplitude at both −120 and 60 mV. On the contrary, hTRAAK cells revealed a sustained enhancement of currents coherently with the expression of hTRAAK channels.

**Figure 1:**
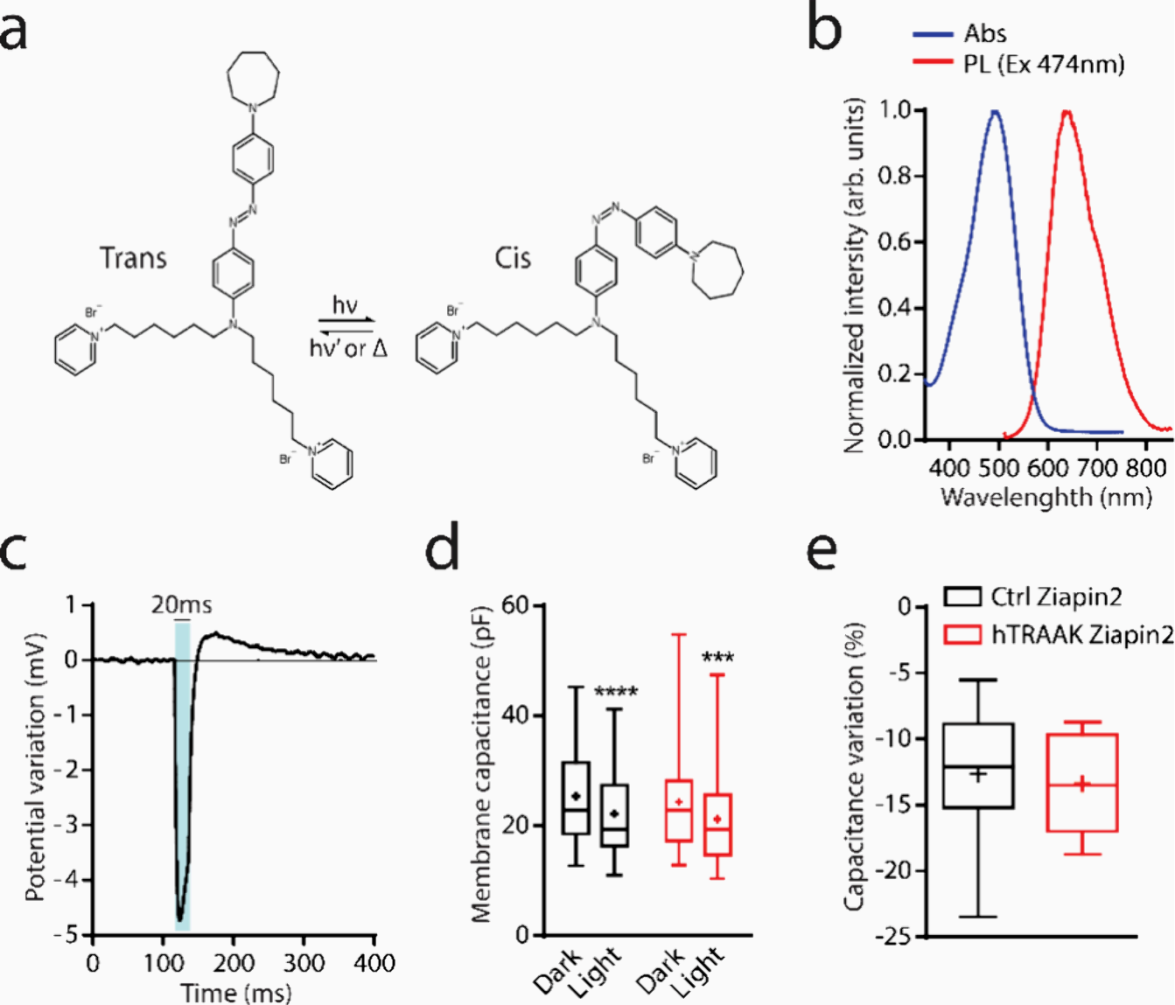
hTRAAK overexpression does not affect Ziapin2 capability of modulating membrane capacitance. (a) Chemical formulas of Ziapin2 in both *trans* and *cis* configuration. (b) Absorption (blue) and PL (red) spectra acquired with fixed excitation at 474 nm and normalized to emission maximum. (c) Representative trace of photomodulation of membrane potential in HEK293T cells loaded with 25 μM of Ziapin2 and illuminated for 20 ms with visible light at 54 mW/mm^2^. (d) Box plots representing membrane capacitance of both untransfected (*Ctrl*) and pIRES:hTRAAK transfected (*hTRAAK*) cells before and during a 200 ms illumination with visible light at 54 mW/mm^2^. Paired Student *t* test/Wilcoxon test; ***p < 0.001, ****p < 0.0001 (Dark vs light). Mann–Whitney *U* test; *p* > 0.05 (Ctrl Ziapin2 dark vs hTRAAK Ziapin2 dark). (e) Plots representing membrane capacitance variation upon light irradiation in both untransfected (*Ctrl*) and pIRES:hTRAAK transfected (*hTRAAK*) cells loaded with 25 μM of Ziapin2. Unpaired Student *t* test; *p* > 0.05. *N* = 14 and 13 for Ctrl Ziapin2 and hTRAAK Ziapin2, respectively.

After vector validation, we tested if the exogenous expression of hTRAAK channels alters the Ziapin2 properties (Figure 1). As previously reported, in the dark, Ziapin2 preferentially partitions inside the plasma membrane in the *trans* configuration. The pyridine groups interact noncovalently with the polar head of the phospholipids in both membrane leaflets. This induces the formation of Ziapin2 dimers via backbone interaction, resulting in a consequent thinning of the membrane and an increase in the cell capacitance. The formation of dimers has been predicted as the more probable interaction between different Ziapin2 molecules by molecular dynamics (MD) simulation even at high molecule concentrations. Visible light illumination generates a *trans-*to*-cis* isomerization that leads to the breaking of Ziapin2 dimers (Figure 1a,b). The consequent membrane relaxation translates into a rapid hyperpolarization, followed by a slight depolarization after the end of the stimulus (Figure 1c). This phenomenon has been reported in a variety of different types of cells, including bacteria, HEK293T cells, neurons, and cardiomyocytes. In excitable cells, the membrane potential variation is sufficient to trigger action potentials.^47,48,51,52^

Since Ziapin2 dimerization generates a light reversible thinning of the membrane, we recorded cell membrane capacitance of both Ctrl and hTRAAK cells before and during visible light illumination to reduce the impact of cell intrinsic variability. Cell membrane capacitance was assessed by applying a voltage step (Δ*V*) of 5 mV. The area of the current transient (Δ*Q*) was normalized to the voltage step to obtain an estimation of the membrane capacitance (*C*_m_ = Δ*Q*/Δ*V*). As described in Figure 1d,e, we observed no significant differences in terms of cell membrane capacitance between the two groups under dark conditions. In agreement with previous studies,^47,49^ we also confirm that visible light illumination causes a drop of membrane capacitance related to Ziapin2 dimer breaking and membrane relaxation (Figure 1d). As expected, the percentage of capacitance variation revealed to be independent of hTRAAK presence (Figure 1e).

### Ziapin2 Partitioning Enhances Whole-Cell Currents in the Presence of hTRAAK Channels

**Figure 2:**
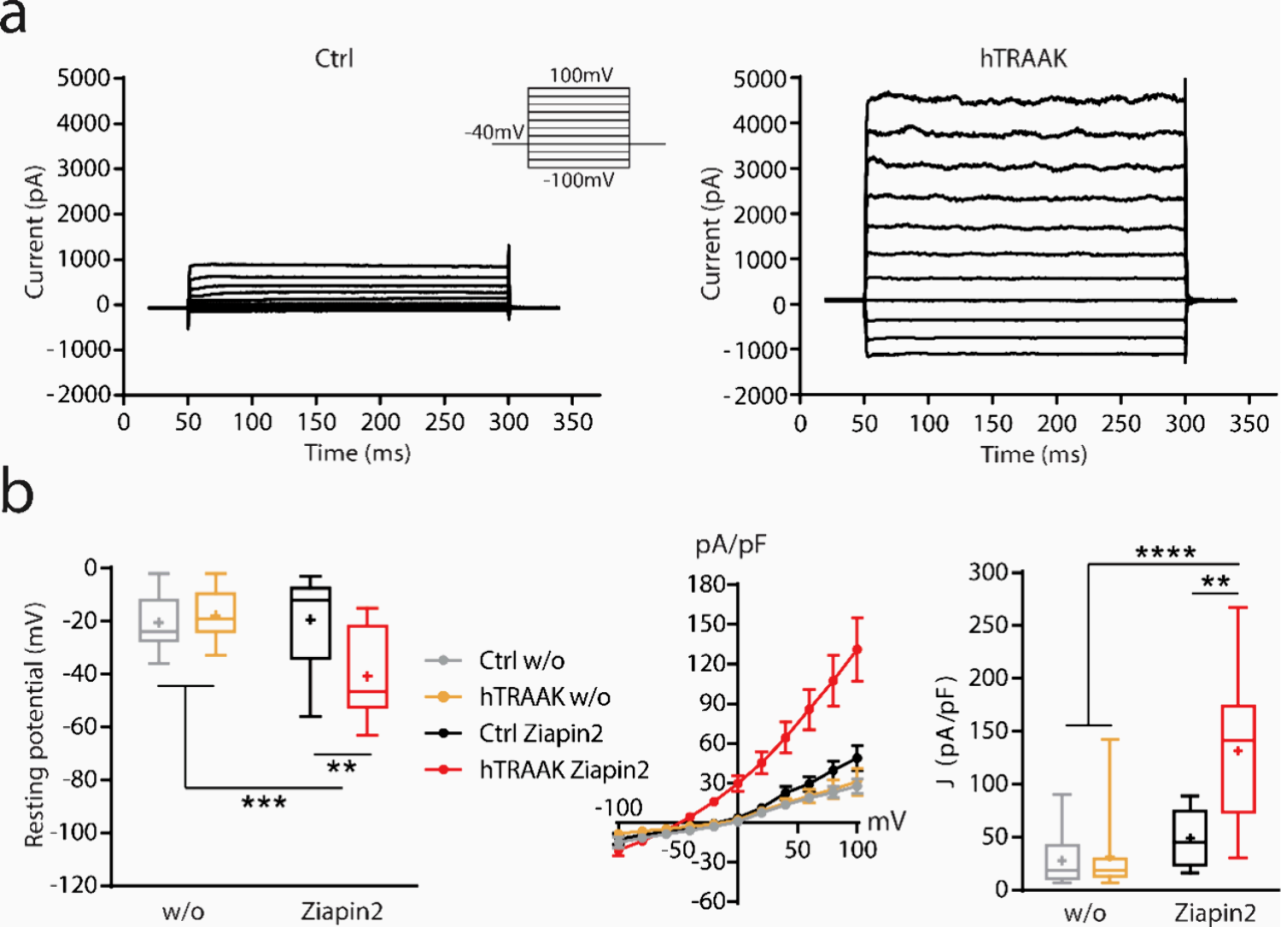
Ziapin2 enhances whole-cell currents in HEK293T cells expressing the hTRAAK channel. (a) Representative traces of whole-cell currents obtained by stimulating both untransfected (*Ctrl*, left) and pIRES:hTRAAK transfected (*hTRAAK*, right) cells with a voltage step protocol from −100 mV to 100 mV; loaded with 25 μM of Ziapin2 and maintained under dark conditions. (b) Plots representing the resting membrane potential (left), current/voltage ratio (*IV* curve, center), and the current density at 100 mV (right) of both untransfected (*Ctrl*) and pIRES:hTRAAK transfected (*hTRAAK*) cells either with or without (w/o) 25 μM of Ziapin2 under dark conditions. Tukey’s multiple comparison test after two-way ANOVA; **p < 0.01, ***p < 0.001, ****p < 0.0001. *N* = 16–17, 13–14, 8–10 and 9–12 for Ctrl w/o, hTRAAK w/o, Ctrl Ziapin2 and hTRAAK Ziapin2, respectively.

Since Ziapin2 generates membrane distortion after internalization in the dark, we evaluated the currents of both Ctrl and hTRAAK cells by voltage-clamp whole cell recordings in the presence or in the absence of the compound (Figure 2). We applied a voltage step protocol to maintain the membrane potential at different voltages (ranging from −100 to 100 mV; Δ*V* = 20 mV) and we recorded the total currents passing through the membrane (Figures 2a and S2). We measured the amplitude of each current (Δ*I*) and normalized it to the cell membrane capacitance (*J* = ΔI/*Cm*).

Ctrl cells, independent on the presence of Ziapin2, were in general characterized by the presence of low amplitude currents at negative voltages (less than −20 mV) and a rapid increase in current density at positive potentials (more than 20 mV), which is coherent with the prevalence of voltage-gated potassium channels expressed in HEK293T cells.^54−56^ These results showed that, in Ctrl cells, Ziapin2 is not able to alter ion channel activity (Figures 2b and S2). Interestingly, HEK293T cells expressing hTRAAK channels showed a significant increase in whole-cell currents in the presence of Ziapin2, particularly at positive potentials (from 40 to 100 mV). This enhancement was accompanied by a significant hyperpolarization of the resting potential (Figure 2b), in line with a previous study in which TRAAK channels were expressed in cos-7 cells. The authors demonstrated that in the presence of TRAAK, the *I*/*V* relationship is characterized by an evident outward rectification at low extracellular K^+^ concentrations.^16,57^ In addition, in the absence of Ziapin2, hTRAAK cells revealed no significant changes in both whole-cell currents and resting membrane potential (Figure 2b). This phenomenon is in accordance with previous studies reporting that, in resting conditions (in the absence of a mechanical stimulation), TRAAK channels show an open probability of less than 1%.^23^

**Figure 3:**
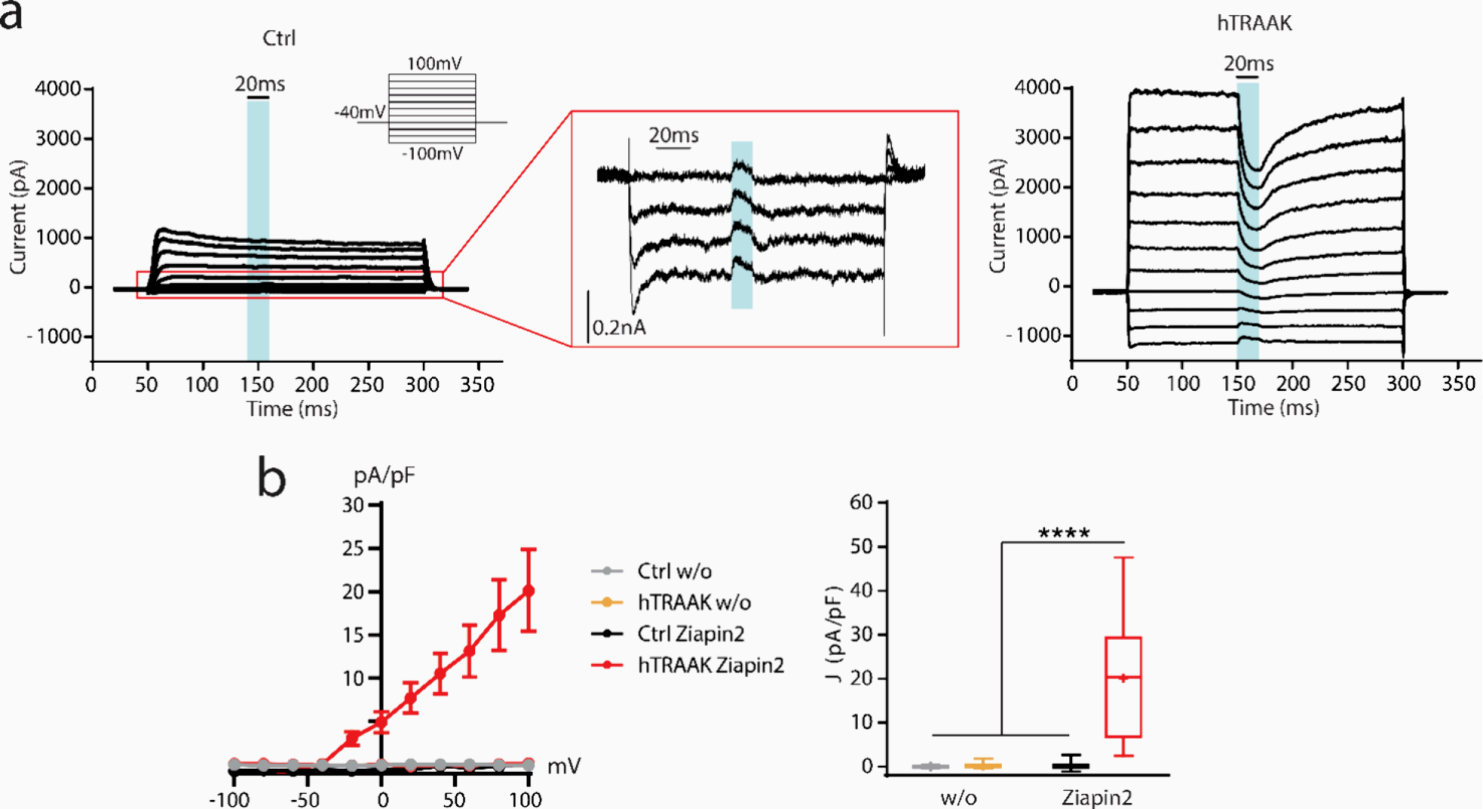
Illuminating Ziapin2 with visible light generates an inward current in HEK293T cells expressing the hTRAAK channel. (a) Representative traces of whole-cell currents obtained by stimulating both untransfected (*Ctrl*, left) and pIRES:hTRAAK transfected (*hTRAAK*, right) cells with a voltage step protocol from −100 mV to 100 mV. Cells were loaded with 25 μM of Ziapin2. During every step, cells were stimulated with visible light for 20 ms at 54 mW/mm^2^. Higher magnification of the voltage step protocol of *Ctrl* cells ranging from −100 mV to −40 mV (center). (b) Plots representing the light-dependent current density variation/voltage ratio (left) and current density variation at 100 mV (right) of both untransfected (*Ctrl*) and pIRES:hTRAAK transfected (*hTRAAK*) cells either with or without (w/o) 25 μM of Ziapin2 under dark conditions. Tukey’s multiple comparison test after two-way ANOVA; ****p < 0.0001. *N* = 17, 13, 8, and 9 for Ctrl w/o, hTRAAK w/o, Ctrl Ziapin2, and hTRAAK Ziapin2, respectively.

We then proceeded to study Ziapin2-mediated optical modulation on HEK293T cells (Figure 3). Upon illumination, Ziapin2 is able to generate a small capacitive photocurrent starting from −100 mV and constantly decreasing its amplitude, until almost disappearing at 100 mV.^47^ In the presence of hTRAAK channels, we observed again a slight current evoked at lower membrane potential; however, starting from −40 mV, a clear inward photocurrent is generated (Figure 3a). This current dramatically increased in terms of amplitude until 100 mV (Figure 3b). In the absence of Ziapin2, no currents were evoked by light stimulation in both Ctrl and hTRAAK cells (Figures 3b and S3).

### Visible Light Illumination Generates a Sustained Depolarization in Cells Expressing hTRAAK Channels

**Figure 4:**
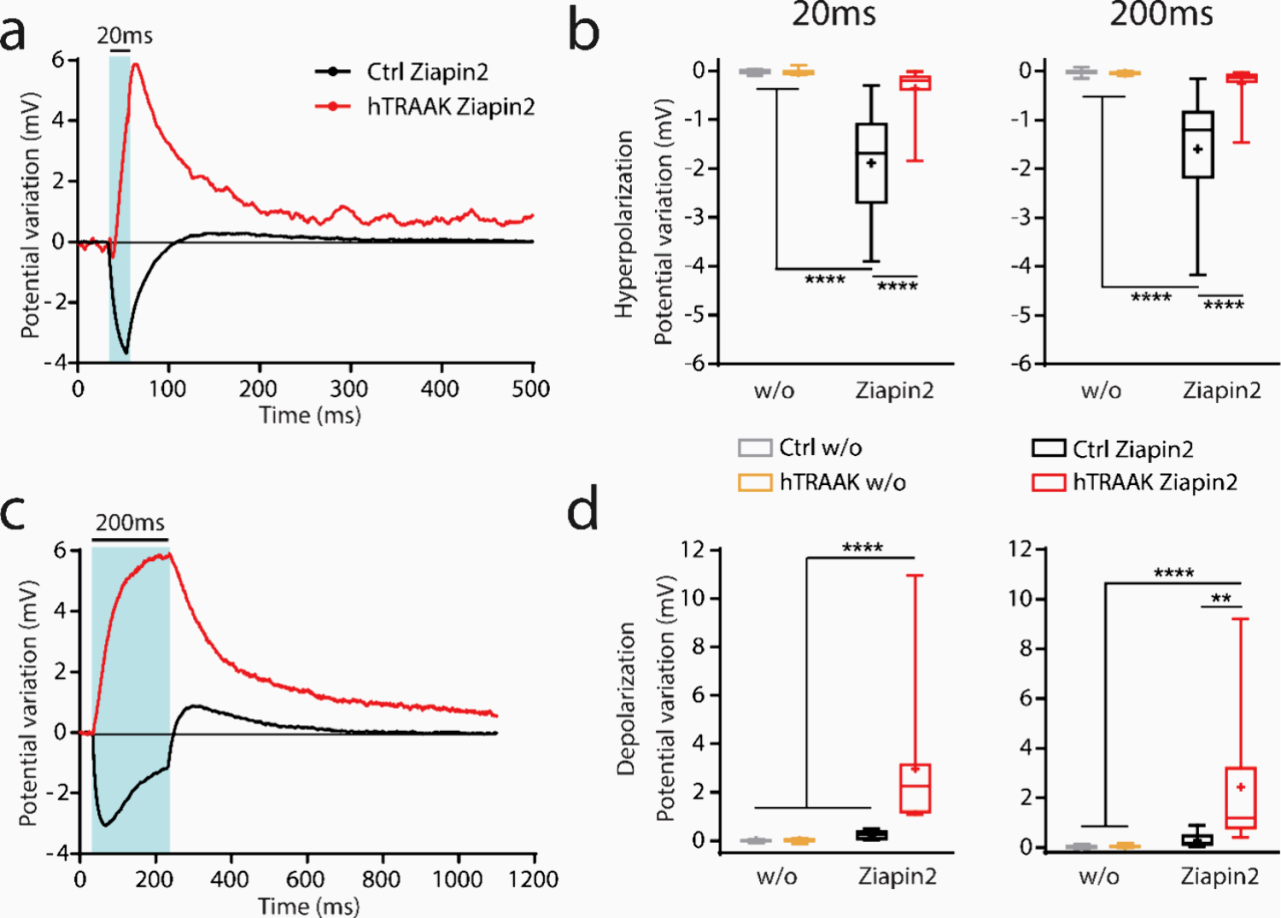
Ziapin2 induces a significant depolarization of HEK293T cells expressing the hTRAAK channel upon light stimulation. (a–c) Representative whole-cell current clamp traces recorded from both untransfected (*Ctrl*, black lines) and pIRES:hTRAAK transfected (*hTRAAK*, red line*s*) cells loaded with 25 μM of Ziapin2 and illuminated at 54 mW/mm^2^ for 20 ms (a) or 200 ms (c). (b–d) Box plots representing peak hyperpolarization (b) and depolarization (d) in both untransfected (*Ctrl*) and pIRES:hTRAAK transfected (*hTRAAK*) cells either with or without (*w/o*) 25 μM of Ziapin2 and illuminated at 54 mW/mm^2^ for 20 ms (left) or 200 ms (right). Tukey’s multiple comparison test after two-way ANOVA; **p < 0.01, ****p < 0.0001. *N* = 17, 13–14, 10, and 13 for Ctrl w/o, hTRAAK w/o, Ctrl Ziapin2 and hTRAAK Ziapin2, respectively.

We verified whether the occurrence of an inward photocurrent could affect membrane potential. Both Ctrl and hTRAAK cells loaded with Ziapin2 were investigated with the whole-cell current clamp technique to evaluate quantitatively the membrane potential modulation upon photoisomerization (Figures 4, S4 and S5). We recorded membrane potential under resting conditions stimulated with both short-(20 ms) and long-lasting (200 ms) light pulses at two different power densities (27 and 54 mW/mm^2^). As it was reported previously,^47−49^ Ziapin2 photoisomerization induced a rapid hyperpolarization of the membrane potential followed by slight depolarization after the end of the light stimulus. Interestingly, hTRAAK cells showed a small hyperpolarization at the beginning of the light stimulation that was rapidly covered by strong depolarization. The depolarization persisted during the illumination and decreased after the end of the stimulus, slowly reaching the baseline (Figure 4a,c). In hTRAAK cells, the amplitude of the hyperpolarization/depolarization phases revealed to be the opposite compared to that of Ctrl cells. In fact, the hyperpolarizing phase was significantly impaired, and the depolarizing phase increased. In addition, in Ctrl cells, the hyperpolarization reached the maximum amplitude at the initial 10–20 ms after light stimulus onset and started decaying before the end of the 200 ms lasting stimulus. In hTRAAK-expressing cells, the depolarization occurred independent of the stimulus duration and power density, suggesting the possibility to modulate the depolarization by changing the illumination duration (Figures 4b,d and S5). We performed the same experiments in the absence of the compound, and we confirmed that no significant membrane potential modulation occurred without Ziapin2 (Figures 4b,d, S4 and S5).

**Figure 5:**
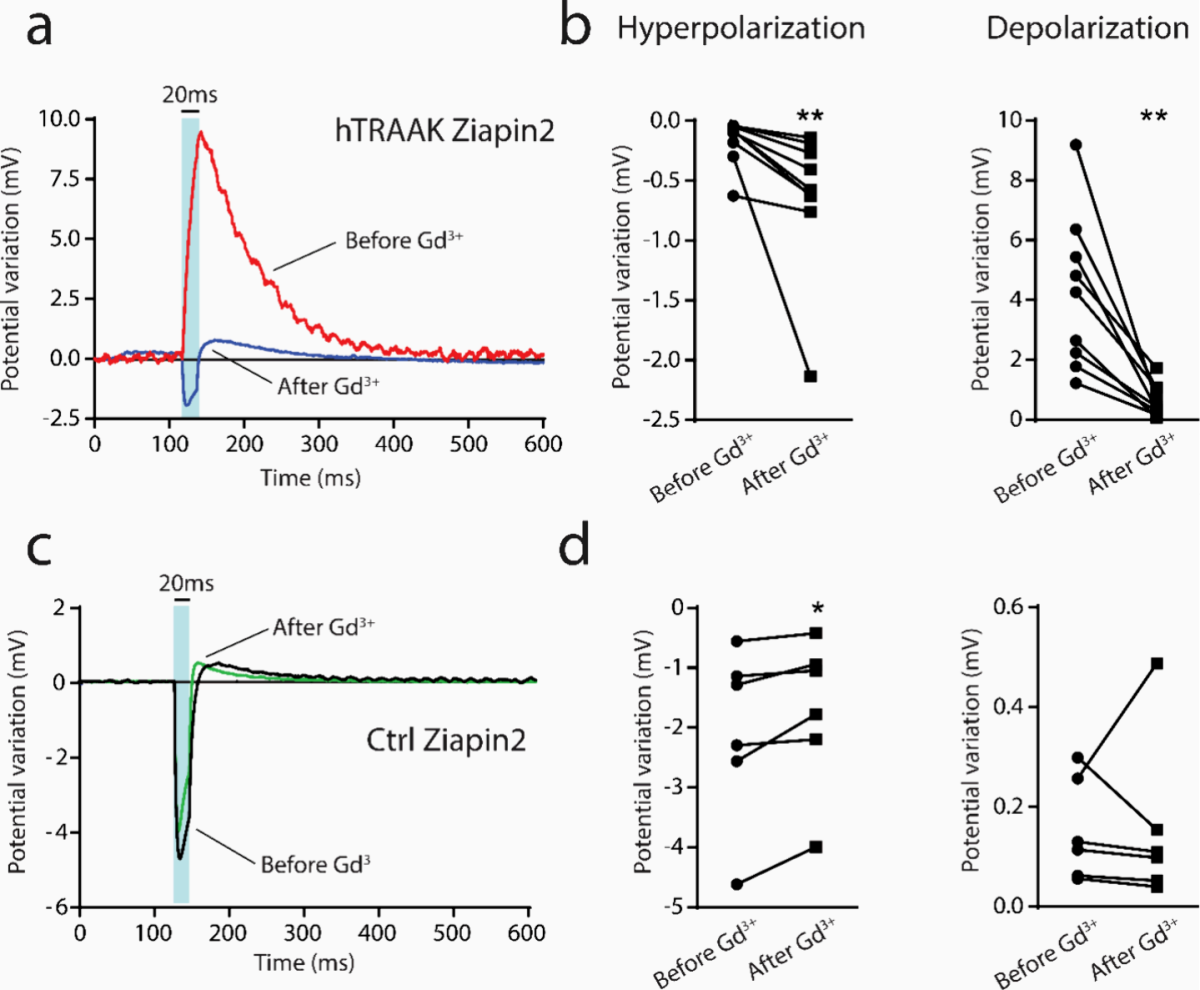
Blockage of hTRAAK opening with gadolinium administration recovers the dynamic of the light-dependent potential modulation. (a–c) Representative whole-cell current clamp traces recorded from both pIRES:hTRAAK transfected (*hTRAAK,* a) and untransfected (*Ctrl;* c) cells loaded with Ziapin2, illuminated at 54 mW/mm^2^ for 20 ms, before (*Before Gd3*^*+*^; red and black line*s*) and after the administration of gadolinium (After *Gd*^*3+*^; blue and green lines). (b–d) Plots representing peak hyperpolarization and depolarization in both pIRES:hTRAAK transfected (*hTRAAK*, b) and untransfected (*Ctrl*, d) cells illuminated at 54 mW/mm^2^ before and after the administration of Gd^3+^. Wilcoxon test; *p < 0.05, **p < 0.01. *N* = 6 and 9 for Ctrl Ziapin2 and hTRAAK Ziapin2, respectively.

To confirm the hypothesis that hTRAAK is responsible for the membrane potential dynamic changes upon illumination, we performed whole-cell current clamp recording in the presence of gadolinium (Gd^3+^). Gd^3+^ is a nonselective blocker that inhibits the opening of mechanosensitive channels.^58,59^ As reported in Figure 5, hTRAAK cells in the presence of traditional extracellular solution depolarize as a response of visible light pulses (see Figure 4). However, after the application of Gd^3+^ (50 μM), light-dependent potential modulation inverted its dynamic. Similar to Ctrl cells, hTRAAK cells treated with Gd^3+^ showed a fast hyperpolarization in concomitance with the light pulse onset followed by a slight depolarization that occurs after the end of the stimulus (Figure 5a). In particular, hTRAAK blockage significantly enhanced the amplitude of the hyperpolarizing phase and extended it for the entire stimulus duration. At the same time, it reduced the depolarizing phase in terms of amplitude and shifted it after the end of the stimulus (Figure 5b). Interestingly, Gd^3+^ was able to partially modify light-dependent potential modulation in Ctrl cells. In fact, even if the dynamic of the potential photomodulation remained unaltered, the hyperpolarizing phase was reduced (Figure 5d). This phenomenon could be ascribed to the mechanism of action of Gd^3+^. It is reported that Gd^3+^ is able to induce a partial rigidification of the plasma membrane and alter phospholipid organization that reduces mechanosensitive channel opening.^59−62^ We previously reported that membrane organization is pivotal in the Ziapin2 photoisomerization process. Indeed, disorganization of the membrane impairs the potential hyperpolarizing phase.^63^

### Proposed Model of Interaction between Ziapin2 and hTRAAK Channels

**Figure 6:**
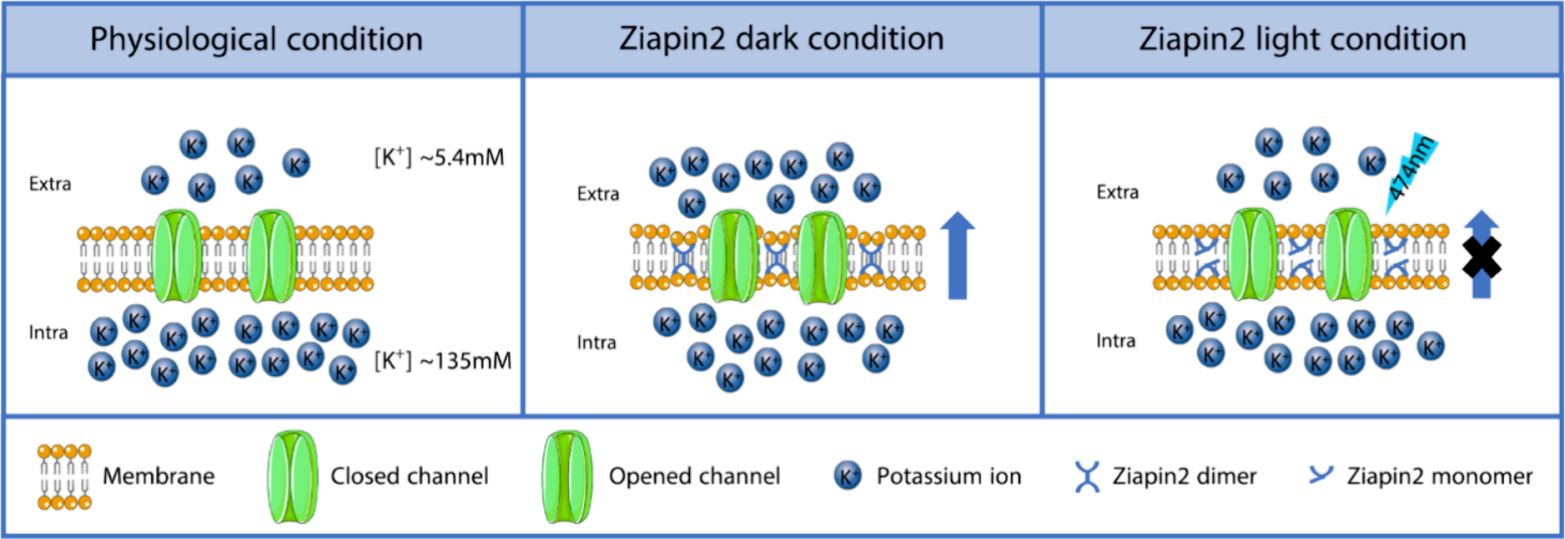
Schematic model of the functional consequences of Ziapin2 partitioning in the cell membrane. Under experimental conditions, HEK293T cells present a significantly higher concentration of K^+^ ions in the intracellular compartment (135 mM) compared to the extracellular environment (5.4 mM). The vast majority of hTRAAK channels remain closed, avoiding the outflux of K^+^. The insertion in the membrane and the consequent formation of Ziapin2 dimers lead to a thinning of the membrane and an increase of the cell capacitance. The membrane stretch caused by Ziapin2 dimerization induces the opening of hTRAAK channels with the consequent generation of a K^+^ outflux. The generation of an outflux of positively charged ions induces a hyperpolarization of the resting potential. Light stimulation triggers Ziapin2 dimer breaking, favoring cell membrane relaxation and hTRAAK channels closing. During illumination, compensatory mechanisms take place to restore physiological resting potential.

We propose here an interpretation of the observed phenomena (Figure 6). According to our setting parameters, K^+^ ion concentration in the extracellular solution is about 5.4 mM and in the intracellular solution 135 mM. As previously reported,^24^ the vast majority of hTRAAK channels remain closed avoiding the outflux of K^+^ or any other ion dislocation. Under dark condition, the *trans*-Ziapin2 forms dimers when partitioned inside the membrane.^47,48^ Dimerization induces consistent thinning of the plasma membrane. We could hypothesize that, during dimerization, the Ziapin2 pyridine group displacement induces a local increase of the membrane surface area that triggers the opening of hTRAAK channels. This swelling-mediated opening of the channels generates a significant K^+^ outflux, experimentally confirmed by the hyperpolarization of the resting potential concomitant with the enhancement of the whole-cell currents. It is important to underline that these results are obtained in not fully physiological conditions. In the experimental model, hTRAAK channels are exogenously overexpressed via transfection, and the concentration of Ziapin2 is high (25 μM). These conditions increase the probability that Ziapin2 and hTRAAK are in close proximity. Illumination with visible light induces a *trans*-to-*cis* isomerization with consequent Ziapin2 dimer breaking and cell membrane relaxation. Accordingly, we could hypothesize that membrane relaxation favors hTRAAK channel closing and K^+^ outflux stop. The depolarization observed during illumination (associated with the generation of an inward current) could be ascribed to the onset of compensatory mechanisms (i.e., ion transporters/pumps) that take place to counterbalance membrane hyperpolarization and recover the physiological K^+^ electrochemical gradient. The recovery of the membrane potential dynamics observed after the addition of Gd^3+^ confirms and corroborates this hypothesis. After the end of the light stimulus, Ziapin2 returns to *trans* configuration and redimerizes stretching again the cell membrane and triggering the progressive reopening of hTRAAK.

## Discussion and Conclusions

K2P are a mechanosensitive family of protein channels that allow for the passive transport of K^+^ ions through the membrane.^64^ In physiological conditions, they are known to be involved in a plethora of different processes: cell volume regulation, membrane potential maintenance, extracellular K^+^ buffering, cell development, and healthiness.^2−5^ They are widely expressed at the level of the central nervous system and regulate action potential maintenance and synaptic transmission.^20,22,23^ K2P channels were revealed to be involved in several pathological conditions, such as depression, pain, and cerebral stroke.^3^ It has recently been reported that K2P channels are overexpressed in rd1 mice that present an early onset of severe retinal degeneration. Some authors justify mechanosensory channel upregulation as a protective mechanism able to induce retinal ganglion cell (RGC) hyperpolarization and prevent excitotoxicity.^27^ For all of these reasons, K2P channels have attracted the interest of the scientific community as an important tool for the modulation of cellular activity and as a possible target for neurodegenerative diseases. In the present work, we demonstrated the possibility of triggering mechanosensory channel opening.

We used a previously well-studied amphiphilic azobenzene derivative, Ziapin2, characterized by noncovalent interactions with the plasma membrane.^47,48^ The insertion of Ziapin2 generates a thinning of the plasma membrane coherent with an increase in the cell capacitance. Contrary to traditional drugs such as small molecules, the mechanism of action of Ziapin2 relies on the capability of altering in a light-dependent manner the cell passive electrical properties without directly affecting biochemical processes and/or cell metabolism. It persists inside the plasma membrane for at least 7 days *in vitro* maintaining its biophysical properties and revealed to be effective also *in vivo* in adult wild-type mice. Ziapin2 does not affect cell viability and does not induce either immunological responses nor gliosis. Our data indicate for the first time that Ziapin2 can also be used as an ion channel modulator. Its capability to stretch the membrane when not exposed to light results to be *per se* sufficient to open mechanosensitive channels. The Ziapin2-mediated opening of mechanosensory channels was revealed to be reversible in a light-dependent manner. Indeed, even a single light pulse is sufficient to trigger Ziapin2 dedimerization, membrane relaxation, and closure of hTRAAK channels. The possibility to induce the opening of mechanosensitive channels without the application of an external mechanical stimulus represents a promising tool for the transient modulation of membrane potential. The opening of mechanosensitive channels and the increase of cell capacitance together could reasonably induce a significant reduction of intrinsic excitability of excitable cells without generating biochemical pathway changes. In addition, Ziapin2 does not interact directly with hTRAAK channels; the opening of the channels is exclusively due to the membrane thinning of Ziapin2 after dimerization, and no allosteric modifications seem involved. This could be ascribed to the nonspecific interaction of Ziapin2 with the plasma membrane. Indeed, the Ziapin2 mechanism of action, characterized by the increasing of cell capacitance and the light-dependent membrane potential hyperpolarization, is generally preserved in every cell type tested. In this regard, we can reasonably hypothesize that it is possible to use Ziapin2 to modulate other mechanosensitive channels permeable to K^+^ like TREK-1, TREK-2 (both related to TRAAK) and even channels permeable to Ca^2+^ such as Piezo1, 2 and TRPV4. However, it is not possible to exclude the involvement of other types of channels (i.e., voltage-gated channels) that could be triggered by the initial change in membrane potential. In fact, in mature and differentiated cells like neurons or cardiomyocytes, the depolarization, which occurs after the initial light-driven hyperpolarization, exceeds in amplitude the simple overshooting expected by the restoration of capacitance. This phenomenon could be explained by taking into consideration the involvement of other types of ion channels not traditionally expressed by immortalized cells. At the same time, HEK293T cells express endogenous mechanosensitive channels (i.e., Piezo1)^65^ that do not seem activated by Ziapin2 in our experimental conditions. We hypothesize that the absence of Piezo1 activation by Ziapin2 could be ascribed to the low expression of the channel.

We reckon that Ziapin2 can be used as light-mechanosensory channel light-driven modulator, which is suitable for many applications ranging from neurodegenerative disease treatment and promotion of cell maturation (i.e., angiogenesis) in the regenerative medicine field to the study of the basic mechanisms of mechanotransduction.^66^ As an example, a possible application could be as a light-modulated drug to compensate for hyperexcitability and possibly revert neuronal physiological alterations induced by excitotoxicity. Contrary to conventional drugs, Ziapin2 is able to alter electrical properties in a reversible manner without directly affecting the biochemistry and metabolism of the cells. It is fully biocompatible even at high concentrations (25 μM), and it does not compromise neuronal cell maturation or wiring or induce inflammatory responses. All these characteristics associated with the lack of specificity render Ziapin2 a promising tool for a plethora of different biomedical applications.^47^

### Material and Methods

#### Cell Culture Maintenance

In vitro electrophysiological experiments were performed using an immortalized cell line HEK293T (Human Embryonic Kidney), purchased from ATCC. HEK293T cells were cultured in T-25 cell culture flasks containing Dulbecco’s Modified Eagle Medium high glucose (DMEM-HG) culture medium, supplemented with 10% heat-inactivated fetal bovine serum (FBS) and 1% GlutaMAX (200 mM). Culture flasks were maintained in a humidified incubator at 37 °C with 5% CO_2_. When at 80% of confluence, cells were enzymatically detached from the flasks with a 1x trypsin-EDTA solution, plated on sterilized substrates, and left to grow for 24 h before transfection. Prior to cell plating, a layer of fibronectin (2 μg/mL in PBS buffer solution) was deposited on the sample surface and incubated for 1 h at 37 °C to promote cellular adhesion. All reagents were purchased from Invitrogen, specified in detail.

#### Cell Transfection

Prior to the electrophysiological experiments, cells were transfected with a pIRES:hTRAAK plasmid purchased from Addgene. pIRES:hTRAAK was a gift from Dan Minor (Addgene plasmid # 133080; http://n2t.net/addgene:133080; RRID:Addgene_133080).^53^ Transfection was performed using the Lipofectamine 3000 reagent (Life Technologies). Before transfection, HEK293T cells were maintained for at least 30 min in DMEM-HG supplemented with 1% GlutaMAX but in the absence of FBS to induce cellular starvation. Cells were then incubated with a cocktail of Lipofectamine 3000 reagent and 1 ng of pIRES:hTRAAK purified plasmid for each sample for 5 h following the traditional procedures. After the end of the transfection, the medium was substituted with DMEM-HG added with FBS. Twenty-four hours after transfection, cells were ready for the electrophysiological experiments.

#### Electrophysiology

Standard patch clamp recordings were performed with an Axopatch 200B (Axon Instruments) coupled to a Nikon Eclipse Ti inverted microscope. HEK293T cells were measured in whole-cell configuration with freshly pulled glass pipettes (4–7 MΩ), filled with the following intracellular solution [mM]: 12 KCl, 125 K-gluconate, 1 MgCl_2_, 0.1 CaCl_2_, 10 EGTA, 10 HEPES, and 10 ATP-Na_2_. The extracellular solution contained [mM] 135 NaCl, 5.4 KCl, 5 HEPES, 10 glucose, 1.8 CaCl_2_, and 1 MgCl_2_. Acquisition was performed with pClamp-10 software (Axon Instruments). Membrane currents were low pass filtered at 2 kHz and digitized with a sampling rate of 10 kHz (Digidata 1440A, Molecular Devices). A cyan LED coupled to the fluorescence port of the microscope and characterized by the maximum emission wavelength at 474 nm provided the excitation light source. The illuminated spot on the sample has an area of 0.23 mm^2^ and a photoexcitation density of 27 and 54 mW/mm^2^, as measured at the output of the microscope objective. Ziapin2 was synthesized according to the procedure reported in a previous study,^49^ and purity was assessed by ^1^H and ^13^C NMR (Bruker ARX400). For all the reported experiments, Ziapin2 was resuspended in Milli-Q water at an initial concentration of 2 mM. Cell membrane capacitance (*Cm*) was measured by applying a voltage step of 5 mV. The capacitance current area (Δ*Q*) was calculated using Origin software. *Cm* was calculated as *Cm* = Δ*Q*/Δ*V*. Light-dependent drop in cell membrane capacitance was measured by illuminating for 200 ms during the 5 mV voltage step application. The *I*/*V* curve was determined by applying a voltage step protocol ranging from −100 mV to 100 mV (Δ*V* = 20 mV). The voltage-dependent currents recorded for each step were normalized to the membrane capacitance to obtain the current density (*J* = Δ*I*/*Cm*).

#### Statistical Analysis

Data are all expressed as box plots. The box plot elements are the following: center line, median (Q2); cross symbol, mean; box limits, 25th (Q1)–75th (Q3) percentiles; the whisker length is determined by the minimum and the maximum value. Normal distribution was assessed using the D’Agostino and Pearson’s normality test. To compare two samples, Student's *t-test* or Mann–Whitney *U* test was used. For multiple variables, the two-way ANOVA test with Tukey’s correction was used. The significance level was preset to *p* < 0.05 for all tests. Statistical analysis was carried out using GraphPad Prism 6 software.

## Data Availability

The data that support the findings of this study are available from the corresponding author upon reasonable request.

## Supporting Information Available

Supporting Information include four supporting figures: The Supporting Information is available free of charge at https://pubs.acs.org/doi/10.1021/acs.jpcb.3c04551.

Validation of the pIRES:hTRAAK construct in HEK293T; representative traces of whole-cell currents in HEK293T cells expressing hTRAAK channel without Ziapin2; representative traces of whole-cell currents in HEK293T cells expressing hTRAAK channel without Ziapin2 upon irradiation with visible light; representative traces of membrane potential modulation in HEK293T cells expressing hTRAAK channel without Ziapin2 upon irradiation with visible light; and box plots representing peak hyperpolarization and depolarization in HEK293T cells expressing hTRAAK channel with or without Ziapin2, illuminated at 27 mW/mm^2^ for 20 ms or 200 ms (PDF)
