# Supplementary material for: Modulation of Mechanosensitive Potassium Channels by a Membrane-targeted Nongenetic Photoswitch
Source: J Phys Chem B. 2023 Oct 10;127(41):8869–78. doi: 10.1021/acs.jpcb.3c04551 (PMC10591468; doi:10.1021/acs.jpcb.3c04551)
Supplement: Supplementary file 1 — jp3c04551_si_001.pdf [file jp3c04551_si_001.pdf]

Supporting information

Modulation of Mechanosensitive Potassium Channels by a Membrane-targeted Non-genetic Photoswitch

Matteo Moschetta[1], Vito Vurro[1,#], Valentina Sesti[1,2], Chiara Bertarelli[1,2], Giuseppe Maria Paternò[1,3] and Guglielmo Lanzani[1,3,*]

[1] Center for Nano Science and Technology, Istituto Italiano di Tecnologia, Via Rubattino, 81, 20134, Milano, Italy

[2] Department of Chemistry, Materials and Chemical Engineering "Giulio Natta", Politecnico di Milano, Piazza Leonardo da Vinci 32, 20133, Milano, Italy

[3] Department of Physics, Politecnico di Milano, Piazza Leonardo da Vinci 32, 20133, Milano, Italy

[#] Present address: Department of Physics and Astronomy "Augusto Righi", Alma Mater Studiorium Università di Bologna, Viale Berti Pichat 6/2, 40127, Bologna, Italy.

[*] Author to whom correspondence should be addressed: guglielmo.lanzani@iit.it

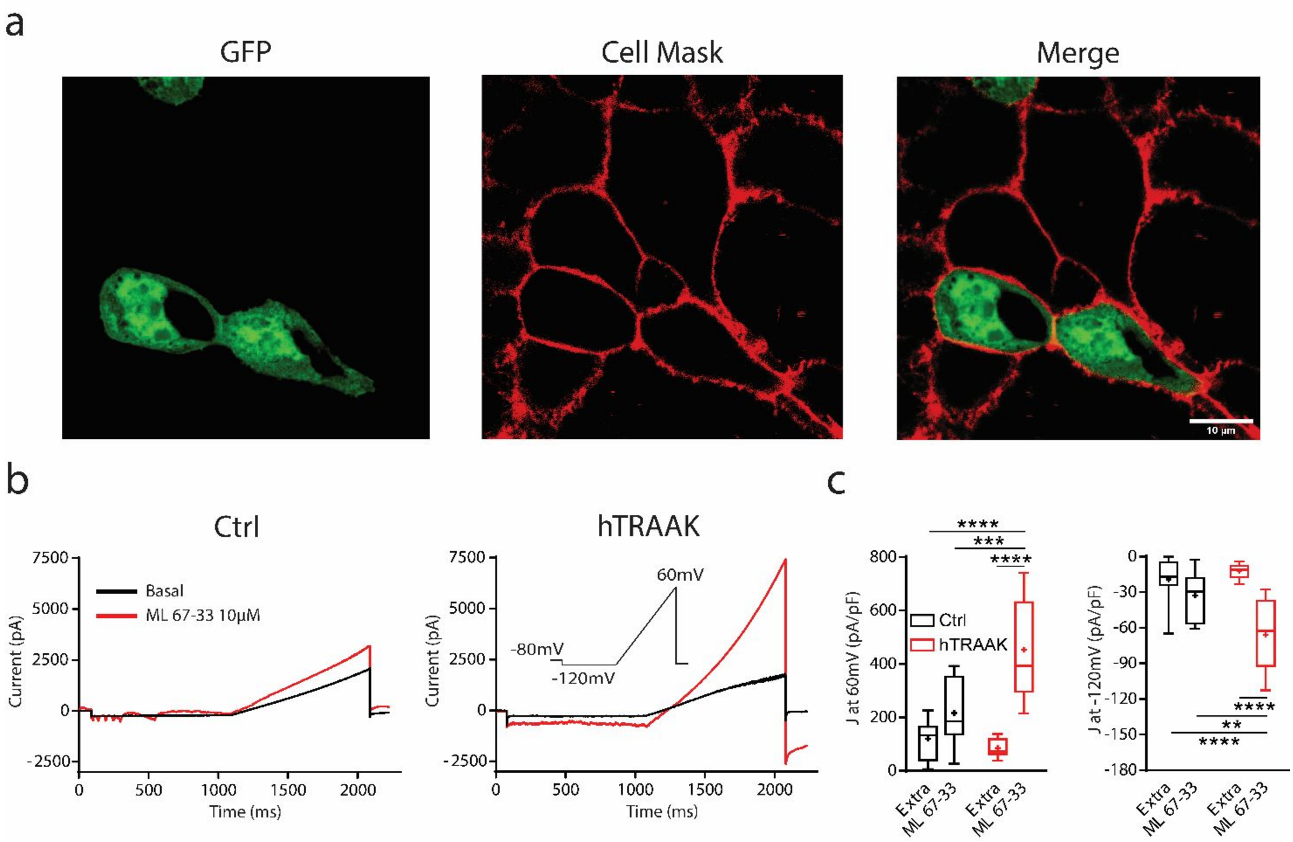

Figure S1. pIRES:hTRAAK validation in HEK293T cells. (a) Representative confocal images of HEK293T cells 24h after transfection with pIRES:hTRAAK. GFP fluorescence in green (*left*) identified transfected cells, cell membrane was stained with CellMask^{TM} in red (*middle*) and merge (*right*). Scale bar: 10µm. (**b**) Representative traces of whole-cell ramp currents obtained by stimulating both untransfected (*Ctrl*) and pIRES:hTRAAK transfected (*hTRAAK*) cells from -120mV to 60mV before (*black lines*) and after (*red lines*) the application of ML 67-33 (10µM). (**c**) Box plots representing current density (J) at 60mV (*left*) and -120mV (*right*). Tukey's multiple comparison test after two-way ANOVA; **p<0.01, ***p<0.001, ****p<0.0001. N=10 and 9 for Ctrl and hTRAAK, respectively.

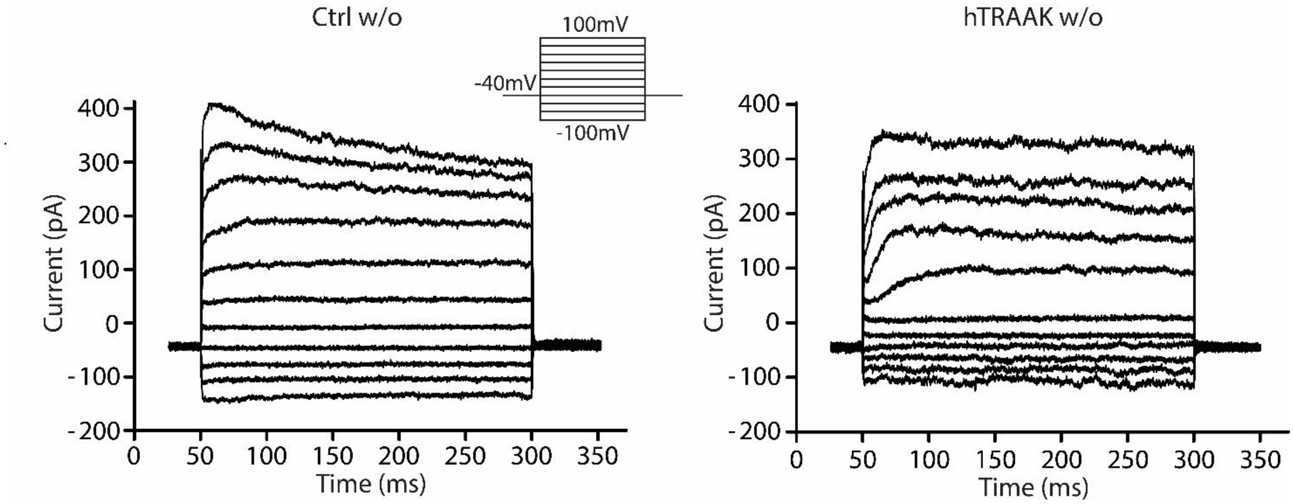

Figure S2. Representative traces of whole-cell currents obtained by stimulating both untransfected (*Ctrl, left*) and pIRES:hTRAAK transfected (*hTRAAK, right*) cells with a voltage step protocol from -100mV to 100mV in absence of Ziapin2.

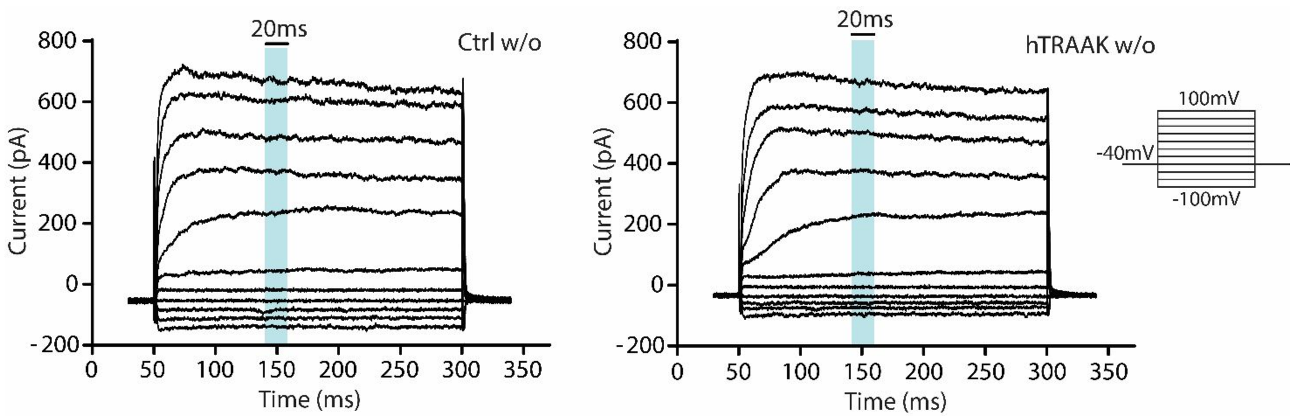

Figure S3. Representative traces of whole-cell currents obtained by stimulating both untransfected (*Ctrl, left*) and pIRES:hTRAAK transfected (*hTRAAK, right*) cells with a voltage step protocol from -100mV to 100mV in absence of Ziapin2. During every step, cells were stimulated with visible light for 20ms at 54mW/mm^2.

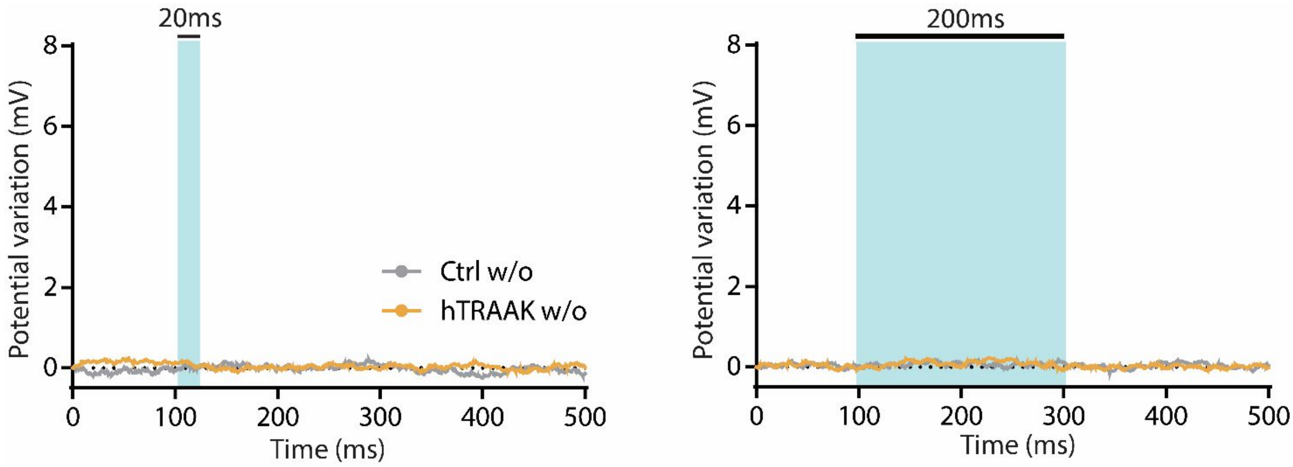

Figure S4. Representative whole cell current clamp traces recorded from both untransfected (*Ctrl, gray lines*) and pIRES:hTRAAK transfected (*hTRAAK, yellow lines*) cells illuminated at 54mW/mm^2 for 20ms (*left*) or 200ms (*right*) in absence of Ziapin2.

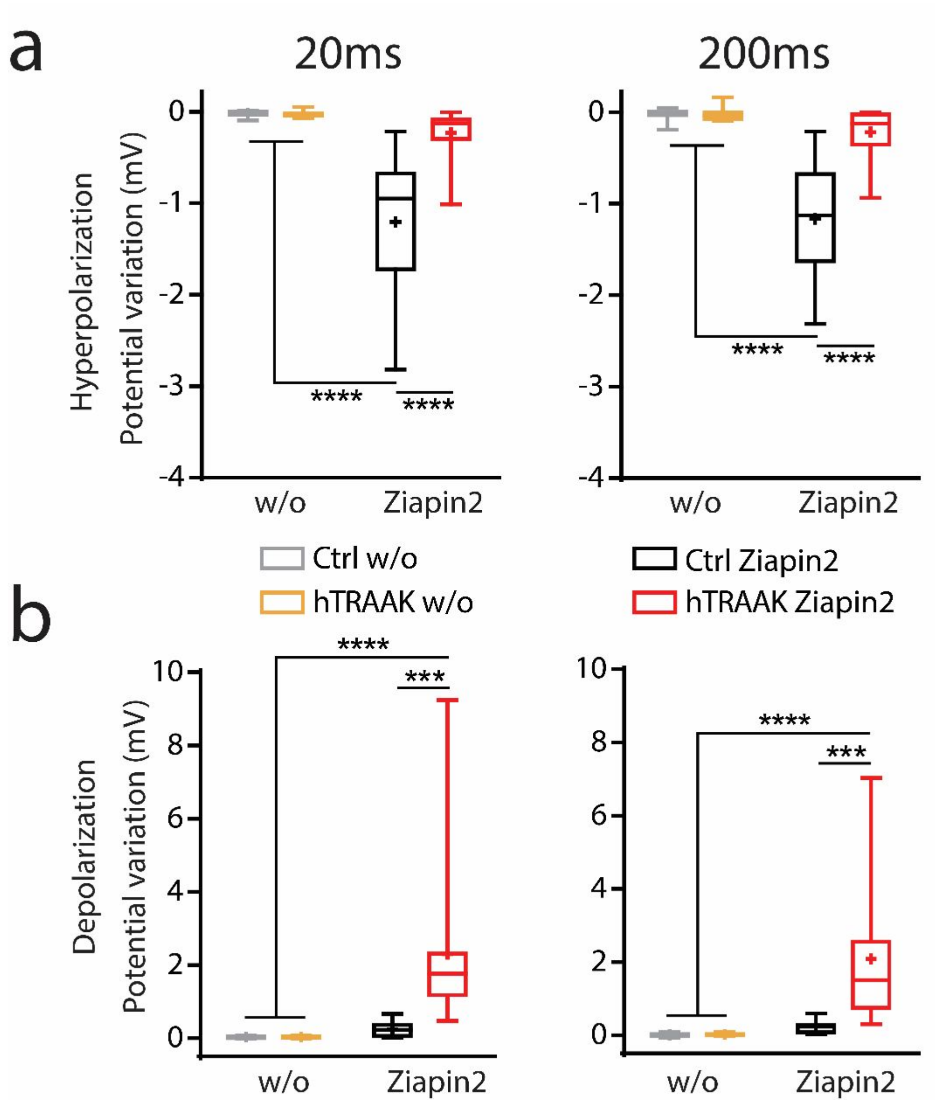

Figure S5. (**a-b**) Box plots representing peak hyperpolarization (**a**) and depolarization (**b**) in both untransfected (*Ctrl*) and pIRES:hTRAAK transfected (*hTRAAK*) cells either with or without (*w/o*) 25µM of Ziapin2 and illuminated at 27mW/mm^2 for 20ms (*left*) or 200ms (*right*). Tukey's multiple comparison test after two-way ANOVA; ***p<0.001, ****p<0.0001. N=17,14, 9-10 and 12-13 for Ctrl w/o, hTRAAK w/o, Ctrl Ziapin2 and hTRAAK Ziapin2, respectively.
